# Polygenic scores and symptom severity change after internet-delivered cognitive behaviour therapy for depression and anxiety

**DOI:** 10.1007/s44192-025-00213-6

**Published:** 2025-06-02

**Authors:** Julia Bäckman, John Wallert, Matthew Halvorsen, James J. Crowley, David Mataix-Cols, Christian Rück

**Affiliations:** 1 Centre for Psychiatry Research, Department of Clinical Neuroscience, Karolinska Institutet, Stockholm Health Care Services, Region Stockholm, Stockholm, Sweden; 2 Department of Genetics, University of North Carolina at Chapel Hill, Chapel Hill, USA; 3 Department of Clinical Sciences, Lund University, Lund, Sweden

## Abstract

Depressive and anxiety disorders are leading causes of disability globally. Therapist-guided, Internet-delivered cognitive behaviour therapy (ICBT) is an established treatment for depression and anxiety, but a considerable proportion of treated patients do not achieve sufficient improvement. Predicting symptom changes based solely on clinical variables is challenging, and genetic data could offer additional insights into who might benefit most from ICBT. We conducted a study of 2,668 adults (62% women, average age 35.6) from the Swedish MULTI-PSYCH cohort, to investigate the association between polygenic scores (PGS) for eight psychiatric and cognitive traits and symptom change after ICBT. All participants had been diagnosed with depression, panic disorder or social anxiety disorder. The primary outcome was a harmonised symptom score. Using large discovery data sets, PGSs were computed for ADHD, autism spectrum disorder, bipolar disorder, major depressive disorder and schizophrenia, cross-disorder psychopathology, educational attainment, and intelligence. Linear mixed-effects models identified a significant negative association between PGS for educational attainment (PGS-EDU) and symptom severity in the adjusted model (B = − 0.69, p = 0.034), suggesting that a higher PGS-EDU was associated with lower symptom severity. There was also a significant PGS-EDU*time interaction, indicating that PGS-EDU also influenced the symptom change rate. When excluding outliers, these effects remained statistically significant. No other PGSs were statistically significant. Sensitivity analysis showed that self-reported education level provided a stronger association to the outcome than PGS-EDU, indicating that the potential clinical applications of PGS are currently limited. While these results await replication, they could have important implications for how the ICBT could be adapted to suit a wider portion of the population.

**Supplementary Information:**

The online version contains supplementary material available at 10.1007/s44192-025-00213-6.

## Introduction

Internet-delivered cognitive behaviour therapy (ICBT) is an effective treatment for anxiety and depressive disorders, with effect sizes comparable to those of in-person CBT [1]. ICBT is an online form of CBT that offers structured programs, exercises, and therapist support, allowing users to access therapy at their own pace and convenience. However, a substantial proportion (10–60%) of patients do not respond sufficiently to treatment [2, 3]. Considering that depressive and anxiety disorders are among the top ten conditions with the highest global burden [4], the impact of psychotherapeutic non-response is substantial, affecting healthcare utilisation, societal costs and overall public health. There is consequently a need to identify predictors of treatment outcome to inform clinical decision-making and allow for better-tailored intervention for these patients. Earlier studies have found a host of predictors for symptom change; baseline symptom severity [2, 5–8], sex [2], education level [5], psychiatric comorbidity, [2], genetic predisposition [9], demographic and socioeconomic indicators, such as age, sex and education level [2, 5, 10]. However, the predictive power of these indicators remain insufficient, and there is a need to search for other possible predictors to reach a level of predictive acuity that is acceptable for use to support clinical decision-making, and tailored intervention.

Using genetic information for prediction has recently emerged as a complement to clinical and demographic variables. Decades of twin, adoption and other family-based studies have established that mental disorders aggregate in families and are substantially heritable [11]. The largest (n cases = 371,184) genome-wide association study (GWAS) of depression identified 243 risk loci associated with the disorder [12]. For anxiety, a handful of loci have been found, likely because the largest GWAS was relatively small (n cases = 25,453, [13, 14]). All psychiatric disorders are polygenic, and the possibility of finding single genetic variants, or single nucleotide polymorphisms (SNPs), with substantial individual effects is consequently limited. Instead, the construction of an aggregated polygenic score (PGS), derived from the weighted sum of many risk alleles of SNPs, constitutes a useful tool for studying the genetics of common psychiatric disorders. PGS for psychiatric disorders represent a portion of unique variance for the target trait and could prove useful for decision-making involving the allocation of patients to genetically informed tailored treatment [15].

Therapygenetics is a relatively new field that aims to identify genetic predictors for psychotherapy outcomes [16]. Some early findings include that higher PGS for autism predicted worse outcomes in patients treated with ICBT for depression [9], that PGS for IQ contributed to prediction of post ICBT symptom severity for depression. Some early findings include that higher PGS for autism predicted worse outcomes in patients treated with ICBT for depression [9], that PGS for IQ contributed to prediction of post ICBT symptom severity for depression [17], in a non-linear ensemble model, and outcome, drop-out and remission status in CBT for dental fear [18]. A PGS for treatment outcome has not yet been developed as the largest GWAS (*n* = 2,724) was underpowered to find SNPs associated with CBT outcome [19]. To increase the sample size, most studies combine data from different sources. This often renders the aggregated sample highly heterogeneous in terms of treatment type, content, dose, and form of delivery [16]. Another way to improve statistical power is to use more homogeneous datasets, i.e., data from highly standardised treatments, such as ICBT, and leveraging repeated weekly symptom measurements instead of only pre-post change in symptoms.

The present study investigated associations of PGS from eight psychiatric and cognitive phenotypes (ADHD, autism, bipolar disorder, depression and schizophrenia, cross-disorder PGS, educational attainment and intelligence) with weekly symptom severity change in a sample of 2,668 adults with anxiety or depression from the MULTI-PSYCH cohort [20] treated with evidence-based ICBT protocols.

## Methods & materials

### Participants

This study was approved by the Regional Ethics Board in Stockholm, Sweden (REPN: 2009/1089–31/2; EPM: 2022–00602-02), and research was carried out following the guidelines of the ethics committee. Informed consent was obtained from all individual participants included in the study. The sample was derived from the MULTI-PSYCH cohort, described in detail in [20]. In summary, participants were recruited between 2009 and 2018 at the Internet Psychiatry Clinic in Stockholm, which specialises in providing ICBT nationwide as part of the public health care system. A total of 2,668 individuals were included in this analysis (depression, n = 1,300, panic disorder (n = 728), or social anxiety disorder, n = 640). See Table 1 for participant characteristics. The sample had 63,4% of individuals with higher education, which is notably high compared to 27% of the Swedish general population in 2016 [21]. Participants that were on psychotropic medication at pre-treatment assessment continued throughout treatment, but medication changes were made if clinically indicated.

**Table 1:** Sociodemographic and clinical characteristics at baseline and post-treatment

	Depression (n = 1300)	Panic disorder (n = 728)	Social anxiety (n = 640)	Total (n = 2668)	Missing
Gender, female	859 (66)	435 (60)	360 (56)	1654 (62)	1 (0)
Age	37.6 (11.9)	34.6 (10.8)	32.7 (10.3)	35.6 (11.4)	
In a relationship	731 (56)	478 (66)	363 (57)	1572 (59)	5 (0)
Children	603 (46)	301 (41)	198 (31)	1102 (41)	5 (0)
Highest education attained					5 (0)
Primary	25 (2)	18 (2)	14 (2)	57 (2)	
Secondary	383 (29)	301 (42)	228 (36)	912 (34)	
Higher	889 (68)	406 (56)	397 (62)	1692 (63)	
Prior inpatient care^a^	91 (7)	74 (10)	27 (4)	192 (7)	115 (4)
Prior suicide attempt^a^	76 (6)	26 (4)	34 (5)	136 (6)	228 (9)
Pre-treatment psychiatric comorbidity^b^	395 (30)	274 (38)	212 (33)	881 (33)	108 (4)
Pre-treatment psychotropic medication^b^	788 (61)	445 (61)	322 (50)	1555 (58)	454 (17)
Number of ICBT modules started^c^	6.90 (3.2)	6.83 (2.9)	7.14 (3.0)	6.94 (3.1)	
MADRS-S pre score	22.7 (6.3)				
MADRS-S post score	13.0 (8.0)				
PDSS-SR pre score		11.0 (4.7)			
PDSS-SR post score		4.98 (4.5)			
LSAS pre score			70.6 (23.5)		
LSAS post score			50.1 (24.1)		

Data are integer count (%) or decimal mean (standard deviation, SD)

*ICBT* Internet-delivered cognitive behaviour therapy, *MADRS-S* Montgomery-Åsberg Depression Rating Scale Self-rated, *PDSS-SR* Panic Disorder Severity Scale—Self Report, *LSAS* Liebowitz Social Anxiety Scale

^a^At any time before treatment start

^b^Assessed at the clinicans visit before treatment start

^c^Total number of modules = 10

### Study design

#### Intervention

The ICBT treatment consisted of 10 treatment modules over 12 weeks for depression [22], panic disorder [23] and social anxiety disorder [24]. All participants were treated at the Internet Psychiatry Clinic in Huddinge, Sweden, which specialises in ICBT. The participants did the treatment on a secure platform, including reading 5–20 pages of text per module, and completing weekly homework assignments. They also communicated with their therapist asynchronously through the platform. The format ensured the treatment content followed protocol and facilitated quality control through automatically distributed questionnaires. For more detail, see [20].

#### Primary outcome measure

Symptom severity was measured weekly from the beginning to end of treatment for the primary outcome using disorder-specific instruments: The Montgomery-Åsberg Depression Rating Scale Self-rated (MADRS-S, [25]) for depression, the Panic Disorder Severity Scale—Self Report (PDSS-SR, [26, 27]) for panic disorder, and the Liebowitz Social Anxiety Scale (LSAS, [28])for social anxiety disorder. All instruments were self-rated and completed via the online platform. To harmonise symptom values across diagnoses, original disorder-specific values were scaled to a common metric (score range 0–100, see details in the Supplement).

#### Genotyping

Genotyping was done in three batches, on either Illumina HumanOmniExpress BeadChips (Illumina, USA) or Infinium Global Screening Array 1.0 BeadArray (Illumina, Inc., San Diego, CA, USA), at the Department of Genomics, Life and Brain Centre, University of Bonn, Germany. More details on genotyping and quality control steps are described in the Supplement.

#### Target dataset

Genotype array data from the target dataset were processed through the Ricopili pipeline v2019_10_15_001 [29]. We first used Ricopili to do pre-imputation quality control on array genotypes across each of the three batches using default thresholds (see Supplement). As part of this, we controlled for cryptic relatedness between samples by removing samples where (1) they had a mean PI_HAT relatedness metric above 0.95, (2) they had evidence of being a duplicated sample based on PI_HAT > 0.95, or (3) they had evidence of cryptic relationship based on PI_HAT > 0.2.

Genotype imputation was performed using Ricopili using impute2 [30] for pre-phasing and minimac3 [31] to impute common variant genotypes along our samples using a 1000 genomes phase 3 reference panel. The cohort was of European ancestry, any non-european sample was excluded. This was done because due to overwhelming focus on European-ancestry individuals in most GWAS done thus far, PGS calculation as it currently stands is more accurate when conducted on European-ancestry subjects. To estimate ancestry, principal component analysis (PCA) was done in EIGENSOFT [32] and a value of > 6 SD from the mean on any of the first three principal components (PCs) was considered outlying and therefore removed. PGS were thereafter calculated with PRS-CS [33] from GWAS summary statistics and samples with European ancestry from the 1000 Genomes Project [34]. PRS-CS is a Bayesian polygenic prediction method that has demonstrated better predictive accuracy than earlier methods [34]. All genetic analyses were conducted using PLINK 1.9 [35].

#### Discovery datasets

Discovery datasets of large GWASs that could be of importance to the outcome and had available summary statistics were used to create individual-level aggregated PGS for each phenotype. See details on n of cases and controls in Table 2. The following discovery datasets were used: ADHD [36], autism spectrum disorder (ASD [37]), bipolar disorder [38], major depressive disorder [39] and schizophrenia [40], cross disorder PGS [41], educational attainment (EDU, [42]) and intelligence IQ, [43]. The cross disorder PGS includes genetic effects of ADHD, ASD, bipolar disorder, major depressive disorder and schizophrenia. The target dataset was not part of these GWAS meta-analyses.

**Table 2:** Discovery datasets used for calculating PGSs, with n case and controls

		N cases	N controls
ADHD	Demontis et al. [36]	20,183	35,191
ASD	Autism Spectrum Disorders Group of the PGC, [37]	16,539	157,234
Bipolar Disorder	Stahl et al. [38]	20,352	31,358
Depression	Wray et al. [39]	135,458	344,901
Schizophrenia	Schizophrenia Working Group of the PGC, [40]	36,989	113,075
Cross-Disorder pathology	Cross-Disorder Group of the PGC [41]	33,332	27,888
Educational attainment	Okbay et al. [42]	101,069	293,723
IQ	Savage et al. [43]	269,867	

*PGS* polygenic score, *ADHD* Attention-Deficit Hyperactivity Disorder, *ASD* Autism Spectrum Disorder

### Statistical analyses

Statistical analysis was done using R [44]. Summary statistics are presented as mean (SD) or count (%) at baseline before ICBT treatment. To estimate the association between PGS and symptom severity change during ICBT treatment, we used linear mixed effects models for repeated measures lme4 package, [45]. Outcome was defined as percentage change of symptom severity based on harmonized scores, as described above. Exposures were defined as the z-transformed values of each of the eight PGSs. To obtain p-values for the coefficients of interest we used the package lmerTest [46], which provides p-values via Satterthwaite’s degrees of freedom method. PGSs were assumed to be normally distributed, and 95% confidence intervals were calculated using the Wald method [47]. Analyses were not adjusted for multiple comparison, as PGSs exhibit genetic correlations, and such adjustments in exploratory studies may be excessively conservative. Unlike GWASs, PGSs aggregate numerous genetic variants for predictive modelling rather than hypothesis testing, reducing the necessity for such adjustments.

### Covariates

We fitted all models using both the linear and quadratic effects of time, which together provided a better fit for data compared to using either one individually, according to Akaike Information Criterion [48]. To account for dropout in our modelling, we incorporated a pattern mixture term by dummy coding participants into two subgroups, non-completers (1) or completers (0); where participants that started < 5 modules were classified as non-completers. ICBT patients could drop out of treatment for reasons related or unrelated to the treatment or symptom change, and reasons are in many cases unknown. By accounting for dropout in our model, we decreased the potential bias of our estimates caused by dropout, which a plain mixed model would otherwise ignore. To control for unwanted genotyping batch-effects, analysis also included three dummies (one per batch). In addition, we controlled for age and sex, which has been suggested as possible predictors of ICBT outcome. We also controlled for ancestry PCs, to account for population stratification. For more details on covariates, see Supplement and Figure S1.

### Modelling procedure

We first estimated the association of time and symptom severity in a model adjusted for time (random slope and intercept), dropout, and batch. For the baseline model, we tested all PGS as fixed effects in separate models only adjusting for time (random slope and intercept), dropout, and batch. For the main analysis, we additionally adjusted for sex, age, and the first five PCs. The interpretation of a significant main effect of a PGS was that the PGS was associated with symptom severity through the treatment period. As a secondary analysis, to investigate the influence of PGS on the rate of change during treatment, we tested for interaction effects (PGS*time) for those PGS that had a significant main effect in the main analysis. A significant PGS*time effect was interpreted as the PGS being associated with the rate of symptom change during the treatment period.

## Results

### Symptom severity

**Fig. 1:**
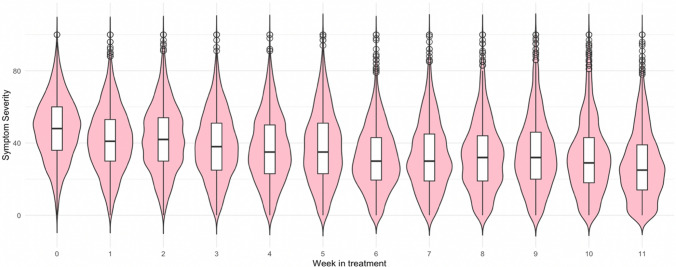
Symptom severity by week in Internet-based Cognitive Behavioural Therapy for the full sample (n = 2668) of participants with depression, panic disorder, or social anxiety disorder. Shapes demonstrate distribution of values, boxes demonstrate spread. Week 0 = baseline, week 11 = post treatment, ¡ = outliers

There was a significant negative main effect of time (fixed) (B =  − 2.47, SE = 0.07, p < 2 × 10−16) on symptom severity, showing the expected reduction in symptom severity during the treatment period, see Fig. 1 and supplementary Figure S2 for each diagnoses separate.

### Symptom severity rating and polygenic polygenic scoresscores

**Fig. 2:**
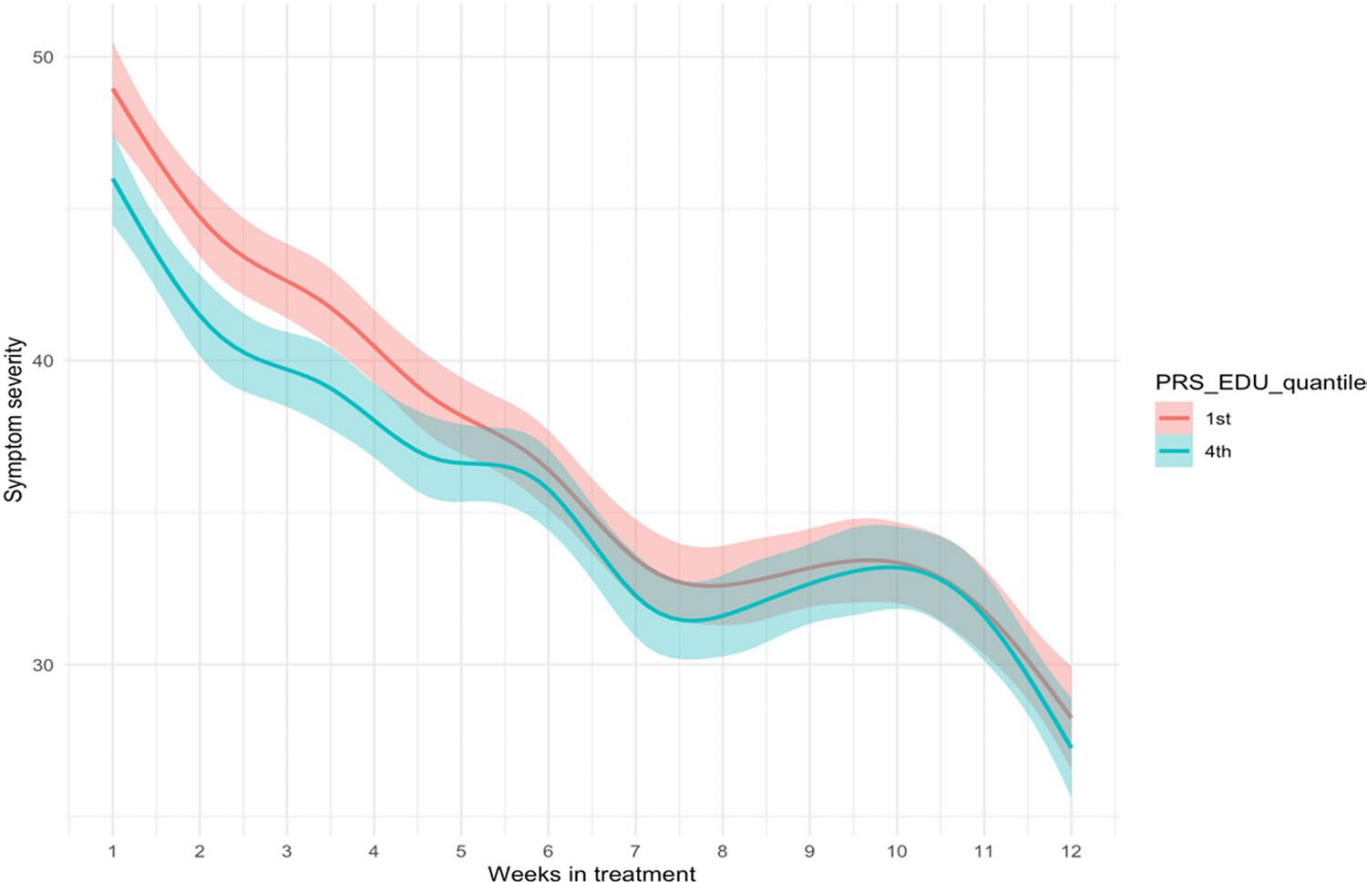
Full sample split into quantiles (n = 667). The figure is showing the estimated slopes for symptom severity change by week in Internet-based Cognitive Behavioural Therapy for first and fourth quantile on the PGS-EDU (i.e. participants with the lowest (red) vs the highest (blue) value on PGS-EDU). The shaded area shows the 95% confidence intervals. Week 1 = baseline, week 12 = post treatment. *PGS-EDU* polygenic score for educational attainment

Results for all models are displayed in Table 3. PGS-EDU had a significant negative association (B = − 0.76, p = 0.02) in the baseline model, which also remained significant when adjusting for age, sex, and PCs in the main model (B = − 0.69, p = 0.04). The interpretation was that an increase of 1 SD change on PGS-EDU was associated with a −0.69 change in symptom severity. To visualize the main results, we split the full sample into quantiles and extracted the first and fourth quantile on the PGS-EDU (i.e. participants with the lowest vs the highest value on PGS-EDU), see Fig. 2. No other PGS showed a significant main effect. The PGS-EDU*time interaction effect was statistically significant in both the baseline and adjusted model (B = 0.07, p = 4.7e-2 vs. B = 0.07, p = 4.5e-2).

**Table 3:** PGS estimates for symptom change

	Baseline				Adjusted			
	Est	SE	p	CI (95%)	Est	SE	p	CI (95%)
Attention-deficit hyperactive disorder	0.16	0.34	0.64	− 0.50, 0.82	0.05	0.34	0.89	− 0.61, 0.71
Autism spectrum disorder	− 0.15	0.34	0.65	− 0.81, 0.51	− 0.22	0.34	0.52	− 0.88, 0.44
Bipolar disorder	− 0.22	0.34	0.50	− 0.88, 0.43	− 0.29	0.34	0.39	− 0.95, 0.37
Major depressive disorder	0.12	0.34	0.72	− 0.54, 0.78	0.01	0.34	0.98	− 0.66, 0.68
Schizophrenia	− 0.30	0.34	0.37	− 0.96, 0.36	− 0.64	0.36	0.08	− 1.34, 0.06
Cross-disorder psychopathology	− 0.45	0.34	0.19	− 1.11, 0.21	− 0.71	0.36	0.05	− 1.41, 0.00
Educational attainment	− 0.76	0.34	0.02*	− 1.42, − 0.10	− 0.69	0.34	0.04*	− 1.35, − 0.03
IQ	− 0.07	0.34	0.84	− 0.73, 0.59	− 0.04	0.34	0.90	− 0.72, 0.63
PGS-EDU*time interaction	0.07	0.04	0.047	0.00, 0.14	0.07	0.04	0.045	0.00, 0.14

All models included time, genotyping batch, and dropout status. Adjusted models included control for age, sex, and the five first principal components for ancestry

*significant < 0.05

*PGS* Polygenic score, *PGS-EDU* Polygenic score for educational attainment. *SE* standard error, *CI* confidence interval

### Sensitivity analyses

We did post hoc outlier analysis to detect influential cases that may have biased our regression estimates on the PGS-EDU model. We used the influence.ME package [49] to calculate Cook’s distance for all observations of all variables (exposure, outcome and covariates). Possible influential observations were identified with a threshold of a Cook’s Distance greater than 3 × the mean, and the analyses were rerun with these outlying observations removed (n = 1,631, total N observations = 32,016). This did not result in altered interpretations of the significant results of the main effect or the interaction effect for PGS-EDU.

An additional post hoc analysis estimated the association between self-reported educational level and symptom severity change during ICBT treatment. The effect was significant, and larger than the main effect both in the baseline model (B = − 2.09, p = 8.15e-15) and the adjusted model (B = − 2.04, p = 4.81e-14), when adding only self-reported educational level to the models. We then fitted the same adjusted model with self-reported education level, but also added PGS-EDU to see if there was a change of model fit. Model fit did not change when adding PGS-EDU (Pseudo-R^2^ (fixed effects) = 0.13), suggesting that the explained variance in the association did not improve when adding the EDU-PGS. However, the correlation between self-reported education level and PGS-EDU was weak (Spearmans rho = 0.1414), indicating that it is not fully overlapping concepts.

## Discussion

ICBT for depression and anxiety disorders is effective, but many patients keep experiencing disabling symptoms after treatment. In this study, we investigated the association between PGS for eight psychiatric and cognitive phenotypes and symptom severity in 2,668 adults with depression, panic disorder, or social anxiety disorder who underwent ICBT. We found that a higher PGS for educational attainment was associated with a higher symptom severity reduction during ICBT. Across different sensitivity analyses, the PGS-EDU association remained significant. In addition, there was a significant PGS-EDU*time interaction, suggesting that PGS-EDU also influenced the rate of change of symptoms during treatment. However, a sensitivity analysis showed that the PGS-EDU did not contribute more than self-reported educational attainment.

There is a resemblance between ICBT and a formal education. Both include shared educational components such as reading, writing, memorising, generalising, application of acquired knowledge and homework assignments. Similar to formal education, ICBT requires that the person takes initiative in planning and carrying out homework exercises. A genetic propensity for educational attainment has previously been associated with cognitive ability, as well as a broader suite of beneficial psychological traits, including self-control and interpersonal skills [50]. Our results suggest that a genetic predisposition for higher educational attainment, could improve the chances of benefitting from ICBT. While, in Sweden, ICBT is available to all citizens regardless of their educational background, we know that, in practice, patients at the Internet Psychiatry Clinic tend to have higher than average educational level (88% with higher education in the sample). A more far-reaching implication of the findings is that ICBT should probably be further adapted to benefit a wider portion of society by, for example, improving the readability of the written materials or replacing a part of the large amount of text with multi-media content.

The sample size was relatively small for a genetic study, which might explain why we were unable to find associations with PGS-IQ even though it has shown a large overlap with PGS-EDU in other studies. A somewhat unexpected finding was the lack of an association between PGS-depression and other psychiatric PGS and symptom change. Despite using a larger sample, we were not able to replicate our previous finding that PGS-ASD was associated with a faster decrease in depression symptom severity [9]. Possible reasons for that include that the sample was still too small to reliably detect associations and the additional cross-disorder heterogeneity in the present larger sample which also included participants with anxiety disorders. Selection bias in the study population may be another contributing factor. The study sample did exhibit selection bias, with participants reporting higher-than-average education levels. This bias may have skewed the sample toward a lower polygenic score (PGS) for both ADHD and ASD, although the actual comorbidity prevalence in the sample was unavailable. Furthermore, the exclusion of participants with schizophrenia could have reduced the overall genetic load for these disorders, potentially influencing the observed associations with symptom change. Finally, it is possible that the genetic risk associated with developing a mental disorder (as captured by PGS) does not necessarily overlap with the genetics of treatment response. The sample size was relatively small for a genetic study, which might explain why we were unable to find associations with PGS-IQ even though it has shown a large overlap with PGS-EDU in other studies. A somewhat unexpected finding was the lack of an association between PGS-depression and other psychiatric PGS and symptom change. Despite using a larger sample, we were not able to replicate our previous finding that PGS-ASD was associated with a faster decrease in depression symptom severity [9]. Possible reasons for that include that the sample was still too small to reliably detect associations and the additional cross-disorder heterogeneity in the present larger sample which also included participants with anxiety disorders. Selection bias in the study population may be another contributing factor. The study sample did exhibit selection bias, with participants reporting higher-than-average education levels. This bias may have skewed the sample toward a lower polygenic score (PGS) for both ADHD and ASD, although the actual comorbidity prevalence in the sample was unavailable. Furthermore, the exclusion of participants with schizophrenia could have reduced the overall genetic load for these disorders, potentially influencing the observed associations with symptom change. Finally, it is possible that the genetic risk associated with developing a mental disorder (as captured by PGS) does not necessarily overlap with the genetics of treatment response.

PGS alone will likely not be used to guide clinical decisions in the future, but could be of use when combined in a multi-PGS approach [51, 52] and/or in combination with other types of data, like clinical risk scores, in order to find individuals at elevated risk for suboptimal outcomes [53]. The utility of PGS in clinical psychiatry should be evaluated in the context of realistic expectations of what PGS can and cannot deliver.

## Strengths and limitations

A strength of this study is the quality of data provided by the highly structured treatment and state-of-the-art clinical routines. All participants were diagnosed by licensed or supervised clinicians in a structured way, providing diagnostic precision. The ICBT treatment format was consistent across individuals and based on gold-standard treatment protocols. Data included weekly symptom severity ratings, which we leveraged using mixed modelling resulting in increased power compared with the more commonly used pre-post-treatment symptom ratings. Another strength was the control for bias from genotype batching and patient dropout. However, there are limitations to consider. The PGS-EDU has a weaker association to symptom severity change than actual attained education and did not add to explained variance when analysed together. To increase sample size and statistical power, we combined data from various clinical diagnoses, which has introduced heterogeneity in the data, although we hold that the larger resulting dataset offsets this limitation, and it also increases generalizability to a larger group of ICBT patients. Another possible limitation is that severe major depression disorder cases were excluded a priori, which means that the results may not generalise to more severe individuals. This further highlights the need for external validation of the present findings as well as caution when extrapolating the results.

## Conclusion

In a large sample of participants with depression and anxiety undergoing ICBT, we found that a higher PGS for educational attainment was associated with a more substantial symptom reduction. However, PGS scores contributed little over and above self-reported educational attainment, suggesting that the potential clinical applications of PGS are still unclear. Regardless, the results could have important implications for how ICBT should be adapted to suit a wider portion of the population by, for example, by limiting the amount of written materials.

## Supplementary Information

Below is the link to the electronic supplementary material.

Supplementary file 1

## Data Availability

The datasets analysed during the current study are not publicly available since dissemination of individual level data is prohibited by Swedish law, but data are available from MULTI-PSYCH on reasonable request.

## Code availability

R code for generating the present results is available upon request.

## References

[1] Hedman-Lagerlöf E, Carlbring P, Svärdman F, Riper H, Cuijpers P, Andersson G. Therapist-supported internet-based cognitive behaviour therapy yields similar effects as face-to-face therapy for psychiatric and somatic disorders: an updated systematic review and meta-analysis. World Psychiatry. 2023;22(2):305–14. 37159350 10.1002/wps.21088 PMC10168168

[2] Rozental A, Andersson G, Carlbring P. In the absence of effects: an individual patient data meta-analysis of non-response and its predictors in internet-based cognitive behavior therapy. Front Psychol. 2019;10:589. 30984061 10.3389/fpsyg.2019.00589 PMC6450428

[3] C. Young, K. Campbell, and C. Dulong, “Internet-Delivered Cognitive Behavioural Therapy for Major Depression and Anxiety Disorders: A Review of Clinical Effectiveness,” in *The Canadian Agency for Drugs and Technologies in Health (CADTH)*, Ottawa, CA: CADTH, 2018, pp. 1–48. 30883066

[4] James SL, Abate D, Abate KH, Abay SM, Abbafati C, Abbasi N, Abbastabar H, Abd-Allah F, Abdela J, Abdelalim A, Abdollahpour I. Global, regional, and national incidence, prevalence, and years lived with disability for 354 diseases and injuries for 195 countries and territories, 1990–2017: a systematic analysis for the Global Burden of Disease Study 2017. Lancet. 2018;392(10159):1789–858. 30496104 10.1016/S0140-6736(18)32279-7 PMC6227754

[5] Edmonds M, Hadjistavropoulos HD, Schneider LH, Dear BF, Titov N. Who benefits most from therapist-assisted internet-delivered cognitive behaviour therapy in clinical practice? Predictors of symptom change and dropout. J Anxiety Disord. 2018;54:24–32. 29421369 10.1016/j.janxdis.2018.01.003

[6] Niles AN, et al. Internet-based cognitive behavior therapy for depression, social anxiety disorder, and panic disorder: effectiveness and predictors of response in a teaching clinic. Behav Res Ther. 2021. 10.1016/j.brat.2020.103767. 33249272 10.1016/j.brat.2020.103767

[7] Hamilton KE, Dobson KS. Cognitive therapy of depression: pretreatment patient predictors of outcome. Clin Psychol Rev. 2002;22(6):875–93. 12214329 10.1016/s0272-7358(02)00106-x

[8] El Alaoui S, Hedman E, Ljótsson B, Lindefors N. Long-term effectiveness and outcome predictors of therapist-guided internet-based cognitive-behavioural therapy for social anxiety disorder in routine psychiatric care. BMJ Open. 2015;5(6): e007902. 26105031 10.1136/bmjopen-2015-007902 PMC4479995

[9] Andersson E, et al. Genetics of response to cognitive behavior therapy in adults with major depression: a preliminary report. Mol Psychiatry. 2019;24(4):484–90. 30410065 10.1038/s41380-018-0289-9 PMC6477793

[10] Karyotaki E, et al. Do guided internet-based interventions result in clinically relevant changes for patients with depression? An individual participant data meta-analysis. Clin Psychol Rev. 2018;63:80–92. 29940401 10.1016/j.cpr.2018.06.007

[11] Smoller JW, Andreassen OA, Edenberg HJ, Faraone SV, Glatt SJ, Kendler KS. Psychiatric genetics and the structure of psychopathology. Mol Psychiatry. 2019;24(3):409–20. 29317742 10.1038/s41380-017-0010-4 PMC6684352

[12] Als TD, et al. Depression pathophysiology, risk prediction of recurrence and comorbid psychiatric disorders using genome-wide analyses. Nat Med. 2023;29(7):1832–44. 37464041 10.1038/s41591-023-02352-1 PMC10839245

[13] van der Walt K, Campbell M, Stein DJ, Dalvie S. Systematic review of genome-wide association studies of anxiety disorders and neuroticism. World J Biol Psychiatry. 2023;24(4):280–91. 35815422 10.1080/15622975.2022.2099970

[14] Purves KL, et al. A major role for common genetic variation in anxiety disorders. Mol Psychiatry. 2020;25(12):3292–303. 31748690 10.1038/s41380-019-0559-1 PMC7237282

[15] Murray GK, Lin T, Austin J, McGrath JJ, Hickie IB, Wray NR. Could polygenic risk scores be useful in psychiatry?: A review. JAMA Psychiat. 2020. 10.1001/jamapsychiatry.2020.3042. 10.1001/jamapsychiatry.2020.3042 33052393

[16] Lester KJ, Eley TC. Therapygenetics: using genetic markers to predict response to psychological treatment for mood and anxiety disorders. Biol Mood Anxiety Disord. 2013;3(1):4. 23388219 10.1186/2045-5380-3-4 PMC3575379

[17] Wallert J, et al. Predicting remission after internet-delivered psychotherapy in patients with depression using machine learning and multi-modal data. Transl Psychiatry. 2022;12(1):357. 36050305 10.1038/s41398-022-02133-3 PMC9437007

[18] Wannemüller A, et al. Genes in treatment: polygenic risk scores for different psychopathologies, neuroticism, educational attainment and IQ and the outcome of two different exposure-based fear treatments. World J Biol Psychiatry. 2021;22(9):699–712. 33970774 10.1080/15622975.2021.1907708

[19] Rayner C, et al. A genome-wide association meta-analysis of prognostic outcomes following cognitive behavioural therapy in individuals with anxiety and depressive disorders. Transl Psychiatry. 2019;9(1):150. 31123309 10.1038/s41398-019-0481-y PMC6533285

[20] Boberg J, et al. Swedish multimodal cohort of patients with anxiety or depression treated with internet-delivered psychotherapy (MULTI-PSYCH). BMJ Open. 2023;13(10): e069427. 37793927 10.1136/bmjopen-2022-069427 PMC10551950

[21] “SCB. Serie Utbildning och forskning,” SCB, une 2017.

[22] Andersson G, Bergström J, Holländare F, Carlbring P, Kaldo V, Ekselius L. Internet-based self-help for depression: randomised controlled trial. Br J Psychiatry. 2005;187:456–61. 16260822 10.1192/bjp.187.5.456

[23] Bergström J, et al. Internet-versus group-administered cognitive behaviour therapy for panic disorder in a psychiatric setting: a randomised trial. BMC Psychiatry. 2010;10(1):54. 20598127 10.1186/1471-244X-10-54 PMC2910662

[24] Hedman E, et al. Internet-based cognitive behavior therapy vs. cognitive behavioral group therapy for social anxiety disorder: a randomized controlled non-inferiority trial. PLoS ONE. 2011;6(3):e18001. 21483704 10.1371/journal.pone.0018001 PMC3070741

[25] Svanborg P, Åsberg M. A new self-rating scale for depression and anxiety states based on the comprehensive psychopathological rating scale. Acta Psychiatr Scand. 1994. 10.1111/j.1600-0447.1994.tb01480.x. 8140903 10.1111/j.1600-0447.1994.tb01480.x

[26] Newman MG, Holmes M, Zuellig AR, Kachin KE, Behar E. The reliability and validity of the panic disorder self-report: a new diagnostic screening measure of panic disorder. Psychol Assess. 2006;18(1):49–61. 16594812 10.1037/1040-3590.18.1.49

[27] Houck PR, Spiegel DA, Shear MK, Rucci P. Reliability of the self-report version of the panic disorder severity scale. Depress Anxiety. 2002;15(4):183–5. 12112724 10.1002/da.10049

[28] Fresco DM, et al. The Liebowitz social anxiety scale: a comparison of the psychometric properties of self-report and clinician-administered formats. Psychol Med. 2001;31(6):1025–35. 11513370 10.1017/s0033291701004056

[29] Lam M, et al. RICOPILI: rapid imputation for COnsortias PIpeLIne. Bioinformatics. 2020;36(3):930–3. 31393554 10.1093/bioinformatics/btz633 PMC7868045

[30] Howie BN, Donnelly P, Marchini J. A flexible and accurate genotype imputation method for the next generation of genome-wide association studies. PLoS Genet. 2009;5(6):e1000529. 19543373 10.1371/journal.pgen.1000529 PMC2689936

[31] Das S, et al. Next-generation genotype imputation service and methods. Nat Genet. 2016;48(10):1284–7. 27571263 10.1038/ng.3656 PMC5157836

[32] Price AL, Patterson NJ, Plenge RM, Weinblatt ME, Shadick NA, Reich D. Principal components analysis corrects for stratification in genome-wide association studies. Nat Genet. 2006;38(8):904–9. 16862161 10.1038/ng1847

[33] Ge T, Chen C-Y, Ni Y, Feng Y-CA, Smoller JW. Polygenic prediction via Bayesian regression and continuous shrinkage priors. Nat Commun. 2019;10(1):1776. 30992449 10.1038/s41467-019-09718-5 PMC6467998

[34] Genomes Project Consortium. A global reference for human genetic variation. Nature. 2015;526(7571):68–74. 26432245 10.1038/nature15393 PMC4750478

[35] Purcell S, et al. PLINK: a tool set for whole-genome association and population-based linkage analyses. Am J Hum Genet. 2007;81(3):559–75. 17701901 10.1086/519795 PMC1950838

[36] Demontis D, et al. Discovery of the first genome-wide significant risk loci for attention deficit/hyperactivity disorder. Nat Genet. 2019;51(1):63–75. 30478444 10.1038/s41588-018-0269-7 PMC6481311

[37] Autism Spectrum Disorders Working Group of The Psychiatric Genomics Consortium. Meta-analysis of GWAS of over 16,000 individuals with autism spectrum disorder highlights a novel locus at 10q24.32 and a significant overlap with schizophrenia. Mol Autism. 2017;8:21. 28540026 10.1186/s13229-017-0137-9 PMC5441062

[38] Stahl EA, et al. Genome-wide association study identifies 30 loci associated with bipolar disorder. Nat Genet. 2019;51(5):793–803. 31043756 10.1038/s41588-019-0397-8 PMC6956732

[39] Wray NR, et al. Genome-wide association analyses identify 44 risk variants and refine the genetic architecture of major depression. Nat Genet. 2018;50(5):668–81. 29700475 10.1038/s41588-018-0090-3 PMC5934326

[40] Schizophrenia Working Group of the Psychiatric Genomics Consortium. Biological insights from 108 schizophrenia-associated genetic loci. Nature. 2014;511(7510):421–7. 25056061 10.1038/nature13595 PMC4112379

[41] Cross-Disorder Group of the Psychiatric Genomics Consortium. Identification of risk loci with shared effects on five major psychiatric disorders: a genome-wide analysis. Lancet. 2013;381(9875):1371–9. 23453885 10.1016/S0140-6736(12)62129-1 PMC3714010

[42] Okbay A, et al. Genome-wide association study identifies 74 loci associated with educational attainment. Nature. 2016;533(7604):539–42. 27225129 10.1038/nature17671 PMC4883595

[43] Savage JE, et al. Genome-wide association meta-analysis in 269,867 individuals identifies new genetic and functional links to intelligence. Nat Genet. 2018;50(7):912–9. 29942086 10.1038/s41588-018-0152-6 PMC6411041

[44] R. C. Team and Others, “R: A language and environment for statistical computing,” 2013.

[45] Bates D, Mächler M, Bolker B, Walker S. Fitting linear mixed-effects models using lme4. J Stat Softw. 2015;67:1–48.

[46] Kuznetsova A, Brockhoff PB, Christensen RHB. lmerTest package: tests in linear mixed effects models. J Stat Softw. 2017;82:1–26.

[47] Wald A. Tests of statistical hypotheses concerning several parameters when the number of observations is large. Trans Am Math Soc. 1943;54(3):426.

[48] Bozdogan H. Model selection and Akaike’s information criterion (AIC): the general theory and its analytical extensions. Psychometrika. 1987;52(3):345–70.

[49] Nieuwenhuis R, Grotenhuis M, Pelzer B. Influence.ME: tools for detecting influential data in mixed effects models. R J. 2012;4(2):38.

[50] Belsky DW, et al. The genetics of success: how single-nucleotide polymorphisms associated with educational attainment relate to life-course development. Psychol Sci. 2016;27(7):957–72. 27251486 10.1177/0956797616643070 PMC4946990

[51] Krapohl E, et al. Multi-polygenic score approach to trait prediction. Mol Psychiatry. 2018;23(5):1368–74. 28785111 10.1038/mp.2017.163 PMC5681246

[52] Albiñana C, et al. Multi-PGS enhances polygenic prediction by combining 937 polygenic scores. Nat Commun. 2023;14(1):4702. 37543680 10.1038/s41467-023-40330-w PMC10404269

[53] Ikeda M, Saito T, Kanazawa T, Iwata N. Polygenic risk score as clinical utility in psychiatry: a clinical viewpoint. J Hum Genet. 2021;66(1):53–60. 32770057 10.1038/s10038-020-0814-y

