# Supplementary material for: Polygenic scores and symptom severity change after internet-delivered cognitive behaviour therapy for depression and anxiety
Source: Discov Ment Health. 2025 Jun 2;5(1):82. doi: 10.1007/s44192-025-00213-6 (PMC12130394; doi:10.1007/s44192-025-00213-6)
Supplement: Supplementary file 1 [file 44192_2025_213_MOESM1_ESM.docx]

## Supplementary methods

### Harmonizing scores

Methods of harmonizing the primary outcome variables within the ICBT data sets to ensure common measurements across treatments: Denoted by (y0-11) the measurements of any given patient at time 0 (baseline), 1-10 (weekly), and 11 (post-treatment). Within each different type of primary outcome measure, we first scaled the patients’ score at baseline (time zero), 0-100. To compute the other timepoints, we used the baseline score and scaled each value accordingly. Finally, we compared the distributions of score for original data and harmonized score for each diagnosis separately to ensure that the data transformation did not result in large discrepancies. For any variable (x), we computed a new variable (z) by normalising it between 0-100.

m <- min(x)

M <- max(x)

z <- (x-m)/(M-m)*100

**Covariates**

Genotyping batch was chosen as covariate to avoid batch effects. Other covariates included sex and age, which has been suggested as possible predictors of symptom changes, and ancestry PCs that could affect the outcome through population stratification. Psychotropic medication status and symptom severity at baseline was on the “causal pathway” from outcome to exposure, and therefore not adjusted for, to avoid overadjustment bias. See **Figure S1** for a directed acyclic graph (DAG) [1] over potential covariates.


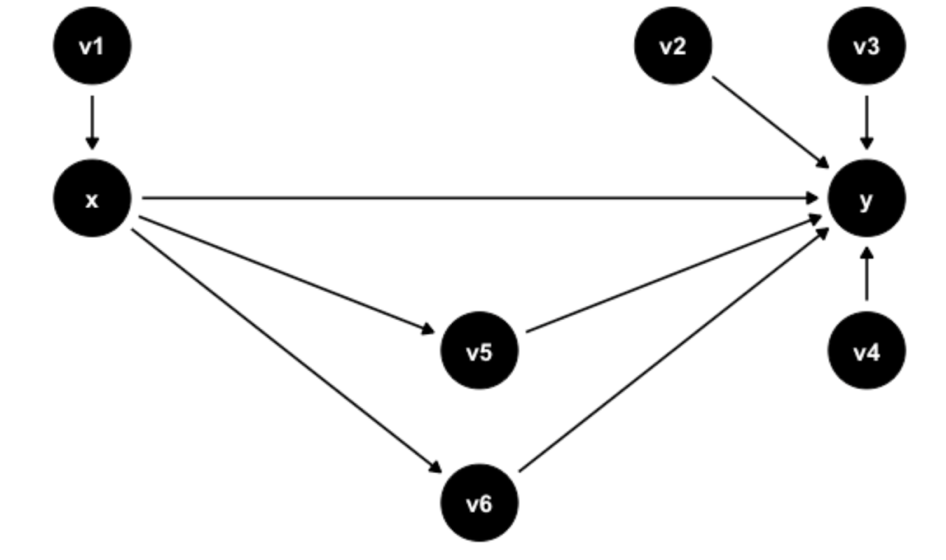


**Figure S1.** Directed acyclic graph (DAG). x=exposure/PGS, y=outcome/symptom change, v1=genotyping batch, v2=age, v3=sex, v4=ancestry PCs, v5= psychotropic medication status, v6=baseline symptom severity.

### Genetic preprocessing

#### Genetic data

We obtained three separate datasets for analysis. The first was a set of 423 individuals diagnosed with panic disorder (PD), genotyped on an illumina CoreExome 12v1 array [2]. The second was a set of 971 individuals with major depressive disorder (MDD), genotyped on an illumina Infinium Global Screening Array 24v1 array [3]. The third (referred to here as the 2019 dataset) was a set of 1900 individuals carrying a mixture of PD, MDD and social anxiety disorder, genotyped on an illumina Infinium Global Screening Array 24v2 array and not previously described in a publication. The total number of samples represented in the raw data is 3294. Reported genders per sample were provided with the PD and MDD datasets, and for the newest dataset were derived by overlaying with the patient medical registry.

#### Dataset cleaning

For each of the three datasets, we performed a within-cohort pruning based on genotype missingness, and then tested for the presence of cryptic relatedness in the data. In each dataset, the subset of variants with missingness across the full set of samples > 0.02 were first identified and removed, and then of the remaining set of variants, the subset of samples with overall missingness > 0.02 were identified and removed. This step left behind 423 samples from PD data, 966 samples from MDD data, and 1891 samples from the 2019 data. On the remaining set of samples and variants in each dataset, we conducted LD pruning in PLINK v1.90b3n using the parameter ‘—indep 50 5 2’, and then used the PLINK ‘—genome’ function on the pruned data to get pairwise relatedness estimates between samples. Across the relatedness data, we proceeded to remove samples that had a mean pi-hat metric with other samples > 0.02. Of the remaining samples, we then scanned for sample comparisons where a pi-hat measurement >0.95 was observed, a pattern consistent with sample duplication. If a duplicate pair was observed in the data, the sample in the pairing that had a higher genotype missingness rate between the two was removed. After this, we scanned remaining sample pairs for instances where the pi-hat measurement was >0.2, consistent with sample cryptic relatedness, and for a given related pair, again pruned the sample in the pairing with a higher genotype missingness rate. Relatedness pruning left us with 421 samples in the PD data, 965 samples in the MDD data, and 1883 samples in the 2019 data.

#### Pre-imputation QC

We used the ‘preimp_dir’ function of ricopili v2019_10_15_001 to perform standard case-only preimputation QC on each of the 3 separate datasets. The pre-imputation QC involves the following, in order: removal of SNPs with call rate < 0.95; removal of samples with call rate across SNPs < 0.98; removal of samples with FHET outside of -0.2 to 0.2; removal of samples with reported/derived gender discordance; removal of samples with ambiguous derived gender; removal of SNPs with a call rate < 0.98; removal of SNPs with hardy-weinberg equilibrium p-value < 1e-6. The pre-imputation QC process left us with 421 samples in PD data, 958 samples in MDD data and 1872 samples in the 2019 data.

#### Imputation

We used the ‘impute_dirsub’ function of ricopili v2019_10_15_001 to impute common variant genotypes along our samples using a 1000 genomes phase 3 reference panel. In our imputation run we used eagle v2.3.5 for pre-phasing, and minimac3 v2.0.1 for imputation. Imputation was conducted on each of the three datasets separately, and for each dataset, led to the production of three different imputation callsets : 1) the ‘bg’ dataset, consisting of SNPs with a P > 0.8 and missingness rate < 0.02; 2) the ‘bgn’ callset, consisting of SNPs with a P > 0.8, INFO > 0.1 and MAF > 0.005; 3) the ‘bgs’ callset, consisting of SNPs with P > 0.8, missingness rate < 0.01 and MAF > 0.05. The bgs callset includes the highest quality imputed SNPs at the cost of a lower total amount captured and are ideal for PGS calculation per sample.

#### Cross-dataset and Ethnicity QC

From the imputed data, we derived a subset of samples across the full data that 1) lacked evidence for cryptic relatedness across the full 3 datasets, and 2) were of most likely European ancestry. For this we first formed a merged dataset across the per-dataset imputed ‘bgs’ callsets, leading to a full dataset consisting of 1,561,953 variant genotype calls across 3269 samples total. We subjected the full merged cohort to the same cryptic relatedness procedure described in the dataset cleaning step and were left with a total of 3233 samples across the full dataset. We next utilized the software package PEDDY v0.4.3 to extract most likely ethnicity classifications across 1000 genomes phase 3 data, to identify the subset of samples that have the classification of EUR. This was done because due to overwhelming focus on European-ancestry individuals in most GWAS done thus far, PGS calculation as it currently stands is more accurate when conducted on European-ancestry subjects. Any sample without a ‘EUR’ classification was excluded from formal PGS comparison. We kept a total of 2932 samples across the full dataset based on these criteria. An additional number of patients were excluded due to missing pretreatment data and a total of 2668 samples were finally included.

Supplementary results

Symptom severity decreased for all groups, regardless of diagnosis (see **Figure S2**).


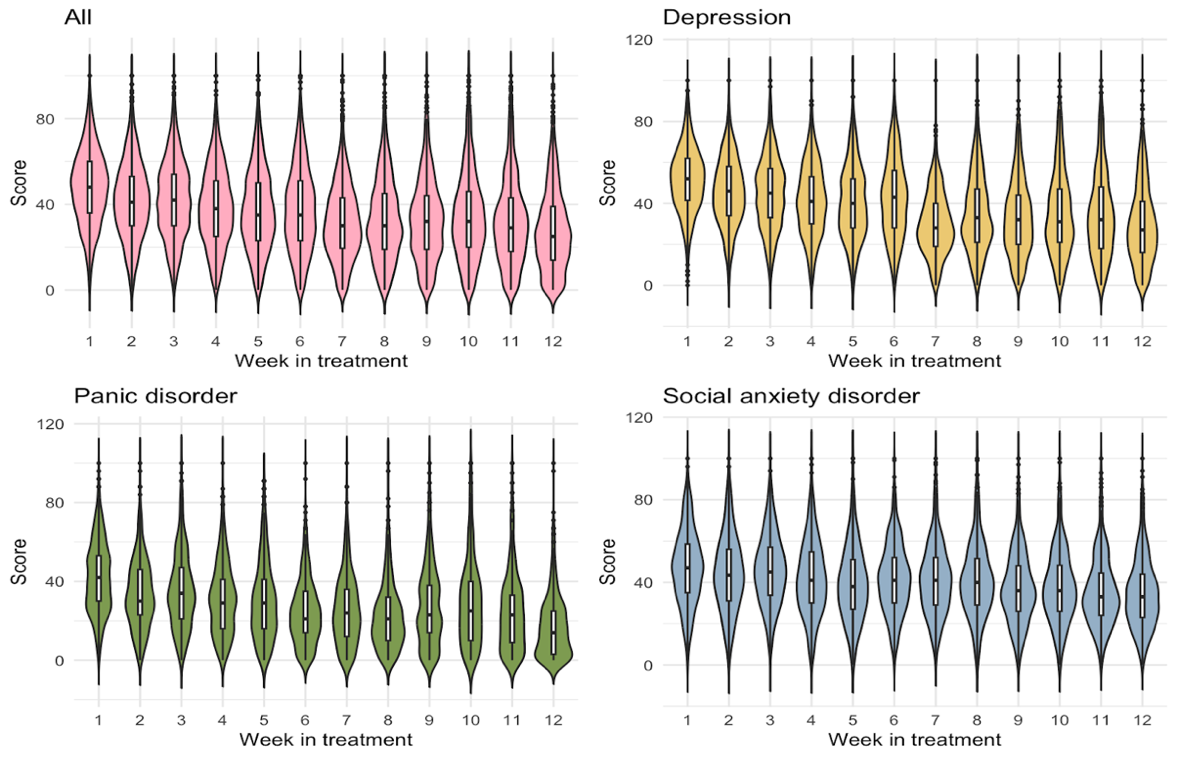


## **Figure S2.** Symptom severity by week in Internet-based Cognitive Behavioral Therapy for the full sample (n=2668), and per diagnosis for depression, panic disorder, or social anxiety disorder. Shapes demonstrate distribution of values, boxes demonstrate spread. Week 0=baseline, week 11=post treatment, ⭘ = outliers.

‡
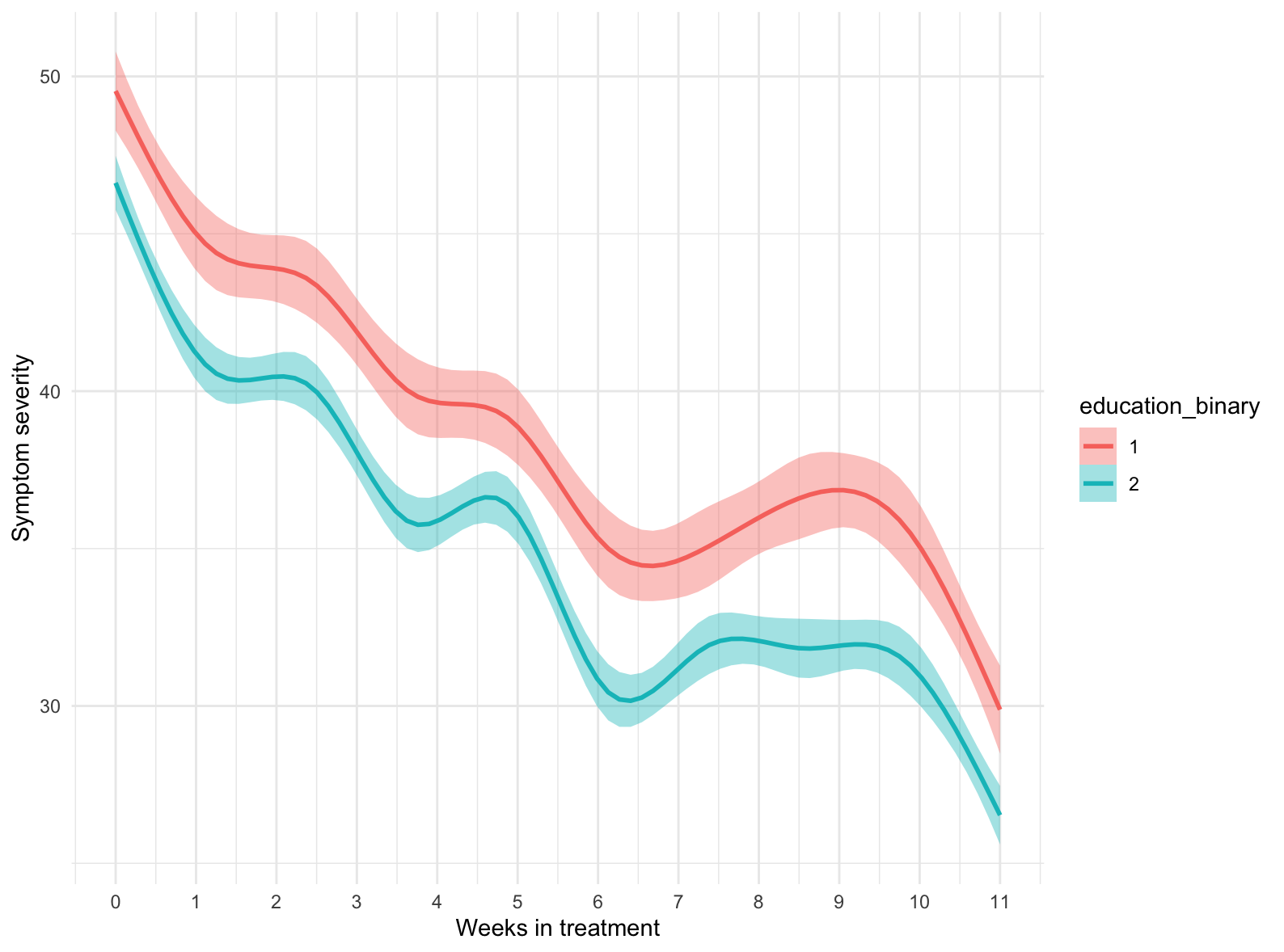


**Figure S3.** Full sample split into low/high education. The figure is showing the estimated slopes for symptom severity change by week in Internet-based Cognitive Behavioural Therapy for participants with low (red) vs high (blue) self-reported education level. The shaded area shows the 95% confidence intervals. Week 0=baseline, week 11=post treatment.

##

## References

[1] K. Thulasiraman and M. N. S. Swamy, “5.7 Acyclic Directed Graphs,” in *Graphs: Theory and Algorithms*, John Wiley and Son, 1992.

[2] A. J. Forstner *et al.*, “Genome-wide association study of panic disorder reveals genetic overlap with neuroticism and depression,” *Mol. Psychiatry*, Nov. 2019.

[3] E. Andersson *et al.*, “Genetics of response to cognitive behavior therapy in adults with major depression: a preliminary report,” *Mol. Psychiatry*, vol. 24, no. 4, pp. 484–490, Apr. 2019.
